# Serological Survey of Foot-and-Mouth Disease Virus in Buffaloes (*Syncerus caffer*) in Zambia

**DOI:** 10.1155/2015/264528

**Published:** 2015-08-05

**Authors:** T. K. W. Sikombe, A. S. Mweene, John Muma, C. Kasanga, Y. Sinkala, F. Banda, M. Mulumba, E. M. Fana, C. Mundia, M. Simuunza

**Affiliations:** ^1^Department of Disease Control, School of Veterinary Medicine, University of Zambia, P.O. Box 32379, Lusaka, Zambia; ^2^Central Veterinary Research Institute, P.O. Box 33980, Lusaka, Zambia; ^3^Faculty of Veterinary Medicine, Sokoine University of Agriculture, P.O. Box 3021, Morogoro, Tanzania; ^4^National Livestock Epidemiology and Information Centre, P.O. Box 30041, Lusaka, Zambia; ^5^Southern African Development Community Secretariat, SADC House, Plot No. 54385, Central Business District, Private Bag 0095, Gaborone, Botswana; ^6^Botswana Vaccine Institute, Private Bag 0031, Gaborone, Botswana; ^7^Department of Veterinary Services, Southern African Development Community, Trans-Boundary Animal Disease Section, Ministry of Agriculture and Livestock, P.O. Box 50060, Lusaka, Zambia

## Abstract

A study was conducted to determine the serotypes of foot-and-mouth disease viruses (FMDV) circulating in African buffaloes (*Syncerus caffer*) from selected areas in Zambia. Sera and probang samples were collected between 2011 and 2012 and analysed for presence of antibodies against FMDV while probang samples were used to isolate the FMDV by observing cytopathic effect (CPE). Samples with CPE were further analysed using antigen ELISA. High FMD seroprevalence was observed and antibodies to all the three Southern African Territories (SAT) serotypes were detected in four study areas represented as follows: SAT2 was 72.7 percent; SAT1 was 62.6 percent; and SAT3 was 26.2 percent. Mixed infections accounted for 68.6 percent of those that were tested positive. For probang samples, CPE were observed in three of the samples, while the antigen ELISA results showed positivity and for SAT1 (*n* = 1) and SAT2 (*n* = 2). It is concluded that FMDV is highly prevalent in Zambian buffaloes which could play an important role in the epidemiology of the disease. Therefore livestock reared at interface with the game parks should be included in all routine FMDV vaccination programmes.

## 1. Introduction

Foot-and-mouth disease (FMD) is a highly infectious viral disease of domestic and wild cloven hoofed animals [1–3]. The disease is caused by the foot-and-mouth disease virus (FMDV) of the genus* Aphthovirus* belonging to the family Picornaviridae. The first report of FMD in Zambia dated from 1933 in Barotseland (now Western Province). Typing of the virus from Zambian FMD outbreaks began in 1948 when the Southern African Territories (SAT) immunological types of FMD virus were recognised [4]. Currently, FMD is endemic in some parts of Northern and Muchinga Provinces along areas bordering Tanzania and in southern border areas between Zambia and Zimbabwe, Botswana and Namibia and along the Kafue and Zambezi flood plains which are also bordered by parts of Kafue National Park. These areas are densely populated with domestic and game animals which are usually in contact for most part of the year. The FMD scenario in Zambia is complicated by the presence of a stable wildlife reservoir, the African buffaloes “*Syncerus caffer*,” and traditional practice of transhumant grazing, where cattle farmers trek their animals to wildlife sanctuaries in search of water and pasture [5–7] and where there are several viruses with high sequence diversity due to the nature of FMDV [8, 9]. African buffaloes are known to be carriers of FMDV and as such contact exposes cattle to the risk of being infected. It has also been reported that FMD may circulate undetected in vaccinated cattle herds and in some indigenous breeds reared in areas where FMD is endemic [10].

Zambia has continued to experience isolated outbreaks of FMD such as those that occurred in Namwala in 2005 [11] and in 2008 [12]; Itezhi-Tezhi in 2006 [13] and in 2008 [12]; and Monze and Mazabuka in 2007 [14] and in 2008 [12]. These outbreaks were considered as reoccurrences of the 2004 SAT1 outbreak (Yona Sinkala, personal communications, 2012). In December 2007, SAT2 FMDV outbreaks occurred in Sesheke district in Western Province and Kazungula district of Southern Province [12]. The disease spilled over in 2008 and spread to Senanga, Mongu, Shang'ombo, and Kalabo districts of Western Province [12]. In 2009, there was an outbreak in Mbala, Northern Province, where SAT1 was isolated [15]. In 2010, there was another outbreak in Mbala district, which spread to Chinsali district, and serotype O was isolated [16]. In 2012, Mbala district and Kazungula/Livingstone experienced further outbreaks, where SAT2 and SAT1 were isolated, respectively [17, 18].

FMD is endemic in Zambia and continues to impact negatively on the livestock industry development. Little understanding of the epidemiology of FMDV has led to the continuous occurrences of the disease in Zambia. Cattle movement and trade restrictions resulting from occurrence of this disease have led to severe negative impacts for pastoral and agropastoral families who are most reliant on livestock products for food and economic security [19]. In addition trade restrictions imposed by other countries mean that the country is not able to participate fully in trade of livestock and its byproducts regionally and internationally.

FMD vaccination campaigns are conducted biannually in most parts of Zambia, where the disease is endemic. Although vaccination offers a potential solution, there are questions surrounding the efficacy of the vaccines used since there are many different serotypes (SAT1, SAT2, and SAT3, type O and type A) of FMD viruses reported to be circulating in Zambia [4, 20]. Due to this the vaccines used may not sufficiently match the field strains circulating that often even their homologous potency is unknown and the cold chain crucial for the success of any FMD vaccination is difficult to maintain. Trivalent vaccines (SAT1, SAT2, and SAT3) were used annually in Southern, Central, and Western Provinces of Zambia before 2006. After 2006, bivalent vaccines (SAT1 and SAT2) were used in Southern, Central, and Western Provinces of Zambia where our study was based, while bivalent vaccines (SAT1 and SAT2 or SAT1 and type A or SAT1 and type O) have been used in Northern and Muchinga Provinces of Zambia, bordering Tanzania; unfortunately this area was not covered by our study. This study therefore was conducted to determine the infection status and FMD virus (FMDV) serotypes circulating in buffaloes in Zambia. Thus the epidemiological situation of FMDV will be discussed.

## 2. Material and Methods

### 2.1. Study Area

**Figure 1:**
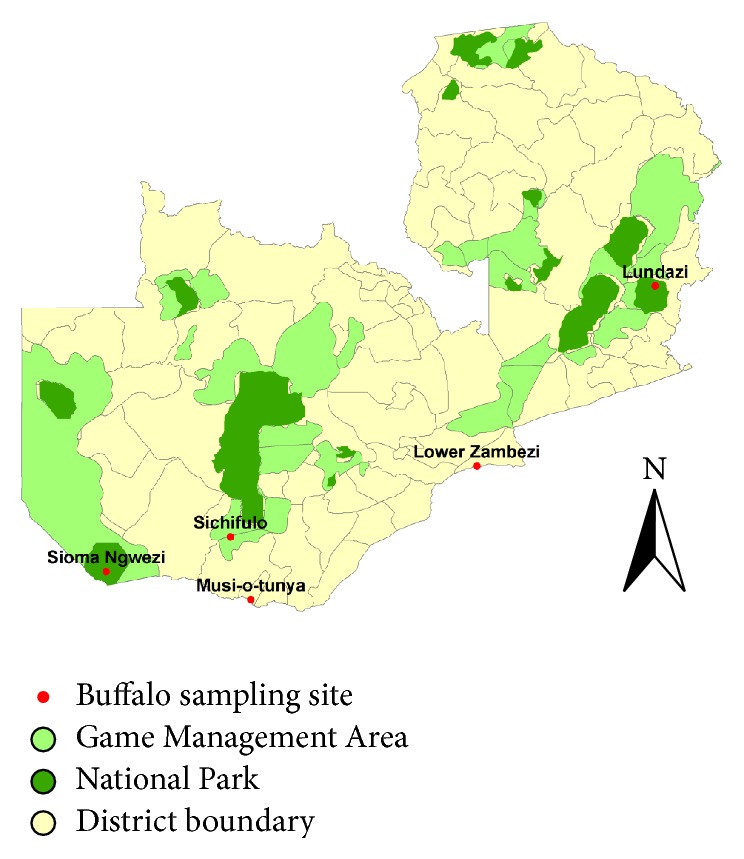
Map of Zambia with sampling sites in Game Management Areas (GMA) and National Parks (NP).

The study was carried out in areas located in National Parks (NP) and Game Management Areas (GMA) in Zambia (Figure 1). Five locations were selected for this study which included Mosi-oa-tunya (S: 17°52.370′; E: 025°50053′), Sichifulo (S: 16°49.470′; E: 025°29.482′), Lower Zambezi (S: 15°38.384′; E: 029°36.756′), Lundazi (S: 12°17.157′; E: 033°10.836′), and Sioma (S: 17°08.8900′; E: 23°65.3367′). These areas were purposively selected because of the presence of interactions between cattle and wildlife resulting from the transhumant cattle husbandry practice where traditional cattle farmers bring their livestock for grazing into wildlife habitats in search of greener pastures and water. These areas are among the major ecosystems of buffaloes in Zambia. Luangwa National Park has the highest density of buffaloes in Zambia (Chuma Simukonda, personal communications, 2011). However, cattle-wildlife interactions are more pronounced in the Kafue flats where FMD outbreaks occur frequently [5].

### 2.2. Study Design

This was a cross-sectional survey carried out from 2011 to 2012 under a special research licence provided by the Zambia Wildlife Authority (ZAWA). It was part of a wider survey undertaken by the Southern African Development Community Transboundary Animal Diseases (SADC TADs) disease surveillance programme. The licence approved sampling of 25 buffaloes from each of the five study areas (Mosi-oa-tunya, Sichifulo, Lower Zambezi, Lundazi, and Sioma) (DVLD/3/22/1: National Parks, Game Reserves and Wildlife). Therefore, we targeted to sample 125 animals.

### 2.3. Sample Collection

Targeted animals were buffaloes aged between six months and six years. This was done to exclude young animals that still had maternal antibodies and those older animals that were no longer prone to infection. The age range of the buffalo was determined by the protrusions of the horns based on aerial view first and before sampling the age range was determined by checking the horns and dentition [21]. Animals were first immobilised through remotely injecting chemical anaesthetic agent, etorphine hydrochloride (M99, Immobilon; Novartis, South Africa). From the immobilised animals, about 8 mL of blood was collected from all the 99 buffaloes through the jugular vein puncture using a sterile vacutainer needle into plain vacutainer tubes. The blood was left to clot overnight at room temperature and then centrifuged at 2500 rpm for 5 minutes to separate the serum. Sera were stored at −20°C until needed for laboratory analysis.

Probang samples were collected from 49 buffaloes using probang cups as recommended in the OIE Terrestrial Manual [22]. The collected probang samples were mixed with FMDV transport media (composed of 0.08 M phosphate buffer containing 0.01% bovine serum albumin, 0.002% phenol red, 1000 units/mL penicillin, 100 units/mL mycostatin, 100 units neomycin, and 50 units/mL polymyxin and adjusted to pH 7.2) [22] in the ratio of 1 : 3 in a conical tube after which the mixture was transferred into a cryotube. The cryotube containing probang sample was then put into a liquid nitrogen tank. Probang cups were disinfected using citric acid (0.2%, wt/vol) and rinsed three times in water and then in PBS between samplings. Probang samples were stored in liquid nitrogen or in the freezer at −70°C in the laboratory awaiting treating and passaging. Furthermore, information on age and sex was recorded and latitude and longitude coordinates were collected with a handheld GPS device (nüvi 205 series; Garmin, USA).

After sampling, the immobilised buffaloes were revived by injection with diprenorphine (M5050, Revivon; Novartis, South Africa). All the samples were collected and processed following World Reference Laboratory (WRL) and World Organisation for Animal Health (Office International des Epizooties (OIE)) guidelines [10].

### 2.4. Sample Analysis

Detection of antibodies against FMDV in sera was done at Central Veterinary Research Institute (CVRI) in Lusaka and Botswana Vaccine Institute (BVI) using the liquid phase blocking ELISA (Institute for Animal Health, Pirbright Laboratory, UK) technique for the detection of antibodies against FMDV in sera as described by [23] and the PrioCHECK FMDV-NS test (Prionics Lelystad B.V., Netherlands), a blocking ELISA that can measure antibody level to 3ABC nonstructural proteins [24].

The probang samples were treated and passaged in RM monolayer cell cultures and then examined for cytopathic effect (CPE). If no CPE was detected after 48 hours, the cells were frozen and thawed, used to inoculate fresh cell cultures, and examined for CPE for another 48 hours. Some field viruses may require several passages before they become adapted [22]. In antigen ELISA (Institute for Animal Health, Pirbright Laboratory, UK) we tested the supernatants of CPE positive cell cultures inoculated with probang samples in order to confirm the specificity of the CPE and to serotype the isolate. The antigen ELISA kit was based on a standard indirect sandwich ELISA technique to determine the presence of FMDV antigens in samples as described by [22].

### 2.5. Data Analysis

Data was stored in basic Excel format for easy handling and storage. The data was transferred to SPSS 16.0 for statistical analysis. Proportion of positive sera, with the 95% confidence intervals (CI) on both LPBE and PrioCHECK FMDV-NS test, were estimated. The associations between categorical variables and the ELISA tests results were evaluated using Fisher's exact test, while Kappa test was used to evaluate the agreement between the LPBE and PrioCHECK FMDV-NS test cross-tabulation results. *p* values <0.05 were considered statistically significant. Spatial mapping of the distribution of FMD in the study areas was done using ArcView_GIS (Environmental Systems Resource Institute, 1992–1999 ArcView 3.2, Redlands, CA).

## 3. Results

### 3.1. Serology Results

#### 3.1.1. LPBE Test

A total of 99 serum samples were tested and the results are shown in Table 1. The overall FMD prevalence based on LPBE SAT serotype results was 92.9 percent (95% CI = 87.8–98.0). The SAT1 prevalence was highest in Lower Zambezi and Lundazi (88.0%, 95% CI = 68.8–97.4), while no animals tested positive to SAT1 serotypes in Sioma National Park. There was a significant difference in SAT1 prevalence among the sampling sites (*p* = 0.001). SAT2 prevalence was highest in Lundazi where all animals tested positive (100%, 95% CI = 83.3–100), with no animals testing positive in Sioma. There was a significant difference in prevalence among the sampling sites (*p* = 0.001). SAT3 prevalence was highest in Sichifulo (50%, 95% CI = 27.2–72.8), with no animals testing positive in Sioma. There was a significant difference in SAT3 prevalence among the sampling sites (*p* = 0.001). All the buffaloes sampled (100%, 95% CI = 83.3–100) from Lower Zambezi and Lundazi were positive to antibodies against FMDV on the LPBE test and all those from Lundazi were positive to at least (100%, 95% CI = 83.3–100) two serotypes.

The few calves (age ranging from six months to eight months) that were sampled were all from Sioma and were all negative for FMDV SAT antibodies. The highest prevalence according to age range was in the 1-2-year category of which all were positive for antibodies against FMDV. In the 3-4-year age category, 93.1% were positive for antibodies against FMDV, while, in the 5-6-year age category, all the samples were positive for FMDV antibodies. There was no significant difference in SAT serotypes prevalence between the different age groups (*p* > 0.05). Similarly, there was no significant difference in the prevalence of SAT serotypes between male and female buffaloes (*p* > 0.05).

#### 3.1.2. PrioCHECK FMDV-NS Test

A total of 99 serum samples were tested on the assay. FMD overall prevalence, based on the PrioCHECK FMDV-NS ELISA test which detects antibodies to nonstructural viral proteins, was high, 84.8% (95% CI = 77.2–91.5). The prevalence according to area of sampling was as follows: Lower Zambezi (*n* = 25), 96% (95% CI = 88.3–103.7); Lundazi (*n* = 25), 100% (95% CI = 100–100); Mosi-oa-tunya (*n* = 25), 80% (95% CI = 64.3–95.7); and Sichifulo (*n* = 20), 75% (95% CI = 56.0–94.0). The few calves (age ranging from six months to eight months) that were sampled were all from Sioma and were all negative for FMDV antibodies on PrioCHECK FMDV-NS test. There was a significant difference in prevalence among the different sampling sites (*p* < 0.05) and this was statistically significant (*p* value = 0.001). Of the 84 buffaloes that tested positive for FMDV NPS antibodies, 69 were strong positives (>70) and 15 were weak positives (>50 and <70). The prevalence according to age categories was also not statistically different (*p* value = 0.413) (Table 2). The overall prevalence of females (*n* = 61) and males (*n* = 38) on PrioCHECK FMDV-NS test was 86.9% (95% CI = 78.4–95.4) and 81.6% (95% CI = 69.3–93.9), respectively. Further, there was no significant difference in the prevalence of antibodies against SAT serotypes tested in LBPE and against nonstructural proteins (PrioCHECK FMDV-NS test) in relation to age and sex (Table 3).

The cross-tabulation of combined test results for the LPBE SAT serotype ELISA and the PrioCHECK FMDV-NS ELISA among the buffaloes sampled in GMA and NP is shown in Table 4. The results showed a fair agreement between results obtained on PrioCHECK FMDV-NS and LPBE SAT serotypes (kappa = 0.296 at 0.001; McNemar = 0.057).

#### 3.1.3. Virus Isolation and Serotyping by Antigen ELISA in Probang Samples

A total of 49 probang samples (Lundazi, *n* = 24, and Lower Zambezi, *n* = 25) were collected, treated, and passaged. Overall cytopathic effects (CPE) suggestive of FMDV replication in primary RM cell cultures were observed in three samples from Lundazi (*n* = 2) and Lower Zambezi (*n* = 1). The CPE was characterised by the fast destruction of the cell monolayer from which infected cells were round and seen singly. Complete destruction of the cell sheet was mostly seen within 48 hours of inoculation of the 1st passage or 2nd passage. Samples with CPE were analysed using the antigen ELISA to identify the serotypes. The antigen ELISA analysis showed that two samples from Lundazi were of SAT2 serotypes, and one sample from Lower Zambezi was of the SAT1 serotype.

## 4. Discussion

The aim of this study was to determine the seroprevalence of the FMD in buffaloes and identify circulating FMDV serotypes in buffalo populations in Zambia. A high prevalence of antibodies against FMDV in buffaloes was observed in all the study areas except in Sioma where only few animals (all calves) were tested. Further, major FMDV SAT types observed to be circulating in buffaloes in Zambia based on the results from LPBE were SAT1, SAT2, and SAT3, while only SAT1 and SAT2 were isolated from probang samples. Our study reveals that FMD could be a problem in study areas. These results corroborate the findings of previous studies which demonstrated high FMDV seroprevalence in buffalo populations in Southern Africa [25, 26]. In our study SAT2 was the most predominant serotype, followed by SAT1 and then SAT3. Overall, all the Game Parks/Game Management Areas had high prevalence of mixed infections, which supports the earlier observation that individual buffaloes may be persistently infected with more than one type of FMDV in the pharyngeal region [27–29]. The LPBE was chosen as it has been used successfully for numerous animal species before, including the African buffalo. While the test shows an almost perfect sensitivity, the specificity in cattle usually is about 95% and similar values were assumed for the African buffalo. However, in cattle up to 18% [30, 31] false positive reactions have been reported. LPBE detects immunoglobulins directed against the capsid or structural proteins of the virus and therefore cannot distinguish antibodies induced by vaccinations using inactivated vaccines from those elicited by infection with live virus [32]. PrioCHECK FMDV-NS detects antibodies against the nonstructural 3ABC protein of FMDV but cannot distinguish between serotypes. However, antibodies to the 3ABC protein are considered to be the most reliable indicators of infection/exposure to FMDV [33, 34]. The specificity of the PrioCHECK FMDV-NS for bovine sera was given as 98.1 percent by [35] while the sensitivity in nonvaccinated, experimentally infected bovines also approached 100 percent. Bronsvoort et al. [36] published sensitivity and specificity estimates of 87.7 percent and 87.3 percent, respectively, for the African buffalo using Bayesian statistics, but their data would be consistent with values closer to those recorded for cattle, in particular if factors like antibody kinetics and sample quality are taken into account.

The study has also revealed that buffaloes within the age of one to two years are most likely to be infected by FMDV as all the buffaloes were positive to FMDV infection at this age. This is in agreement with the findings of previous studies which indicated that, after maternal antibodies wane, the young buffaloes were prone to FMDV infection from the carrier buffaloes [37]. Buffaloes in the age category of 5-6 years were positive for antibodies against FMDV and had mixed infection slightly higher compared to the other categories; this could have been due to the risk that the older the buffaloes are, the more the time they are likely to get infected by different FMDV serotypes is. African buffaloes are efficient maintenance hosts of the SAT type viruses, with an individual animal maintaining the virus up to five years and isolated herds for up to 24 years, although persistence in an individual buffalo is probably not lifelong [37].

Probang samples were collected with the view of isolating FMDV and identifying serotypes in circulation [29, 37, 38]. However, only a small proportion of samples yielded positive results as two, SAT2, and one, SAT1, virus isolates were obtained. This is in line with the report that the excretion of virus by carriers is intermittent [1] and the findings by [10] that reported that the quantity of virus present in the pharynx of carrier animals can vary considerably over time. As part of future studies, it is recommended to obtain more probang samples for virus isolation and also generate nucleotide sequences of these isolates so that the FMDV circulating in these buffaloes can be differentiated to topotypes level to understand their diversity. In addition, the availability of more local FMDV isolates enables the calculation of *r* ^1^ values required to check and possibly adapt preventive and control measures in endemic or epidemic regions where strategic or general vaccination is required with vaccine containing the FMDV subtypes that are active in the area [29].

In this study, based on LPBE, very few SAT3 seropositive buffalo samples were only seropositive for SAT3 (*n* = 5), while slight proportions were SAT1 seropositive only (*n* = 9) or SAT2 seropositive only (*n* = 10). The majority of samples were positive to more than one serotype, and this raises the issue of to what extent the SAT results are cross-reactions [27]. Virus neutralisation assays using local FMDV isolates would be required to further dissect possible cross-reactivities. In addition, the isolation of a SAT3 FMDV from concurrent probang samples would confirm the presence of this serotype in Zambian buffaloes.

The study showed fair agreement between LPBE test and PrioCHECK FMDV-NS test cross-tabulation results. Lack of substantial or perfect agreement could be attributed to the fact that the kinetics and duration of the antibody response to structural and nonstructural viral proteins differ as does the rate of seroconversion [39].

Antibodies to all the three Southern African Territories (SAT) serotypes were detected in buffaloes in the four study areas (Mosi-oa-tunya, Sichifulo, Lower Zambezi, and Lundazi) where age and sex of the buffalo had no effect in FMDV infection/exposure status. This is in agreement with earlier observations in studies conducted in buffaloes in Sub-Saharan Africa [37]. The exception was Sioma where all the four samples collected from buffaloes in age range of six months to eight months were negative for antibodies against FMDV. Therefore no equivocal statement can be made regarding these results as the apparent absence could be attributed to the small sample size and may be that the young buffaloes had not yet been exposed to FMDV.

There is little published on non-SAT serotypes in buffalo in Zambia. From reported outbreaks in Saharan Africa [37], it appears that the majority of outbreaks in the southern regions are due to SAT serotypes with only sporadic introductions of O and A. Zambia being surrounded by other countries which have reported other serotypes cannot be excluded from harbouring other serotypes apart from SAT serotypes due to the reported outbreaks in Kenya (1994 to 2000), Tanzania (1999 to 2000), and Uganda (1995 to 1999) of SAT1, SAT2, and O (as well as A and C in Kenya) [37]. This needs to be looked at in more detail as recent work on sera has shown consistency in results of antibody screening repeatedly over long storage period. The other interesting studies could be comparison of the prevalence of FMDV in buffalo to that in livestock held in Kazungula/Livingstone during the time period of the buffalo sampling as it could possibly shed light on the role that buffaloes have in transmission of the disease to domestic livestock. This is immanent from the fact that the Department of Veterinary Services in the Ministry of Agriculture and Livestock (MAL) had reported FMDV SAT1 outbreaks which belonged to topotype III (WZ) but were not closely related to other SAT1 viruses [17].

It is known that buffaloes play an important role in maintaining FMD infections and are able to infect other susceptible species in Sub-Saharan Africa [26, 40] and that buffaloes have been shown to be the source of infection for impala and domestic animals in proximity of the Kruger National Park (KNP) and other game parks in Southern Africa [40]. References [41–43] demonstrated natural and experimental transmission from carrier buffalo to cattle. However, it should be noted that even though transmission has been demonstrated, the transmission conditions from carrier buffalo are not well understood and difficult to replicate because many attempts at carrying out transmission from carrier buffalo to naive buffalo or cattle have failed, even under conditions of immunosuppression or coinfection with rinderpest and bovine herpes-1 virus [28, 44–47].

## 5. Conclusion

In conclusion, our study has demonstrated high FMDV seroprevalence in buffaloes in Zambia and characterised the SAT serotypes circulating in the country. These findings will play a role in the control of FMD in Zambia because knowledge of circulating FMDV is critical in vaccine matching, which is necessary to ensure vaccine efficacy. Most parts of Zambia are endemic to FMD; therefore strategic or general vaccination is required with vaccine containing the FMDV subtypes that are active in the area [29].

There is still need for molecular characterisation of the positive virus samples on antigen ELISA at the same time antigen titrations should also be performed and *r* ^1^ values should be determined to enable matching with the FMD.

## Figures and Tables

**Table 1:** Seroprevalence of FMDV by LPBE SAT.

Study area NP/GMA	Number tested	SAT serotype			Overall prevalence %	Mixed infection %
		SAT1 %	SAT2 %	SAT3 %		
Lower Zambezi	25	88.0 (68.8–97.4)	84.0 (63.9–95.5)	8.0 (0.98–26)	100.0 (83.3–100)	84.0 (63.9–95.5)

Lundazi	25	88.0 (68.8–97.4)	100.0 (83.3–100)	12.0 (2.5–31.2)	100.0 (83.3–100)	100.0 (83.3–100)

Mosi-oa-tunya	25	32.0 (14.9–53.5)	76.0 (59.3–92.7)	44.0 (24.4–65.1)	92.0 (74.0–99.9)	60.0 (38.7–78.9)

Sichifulo	20	45.0 (23.1–68.3)	35.0 (15.4–59.2)	50.0 (27.2–72.8)	95.0 (75.1–99.9)	35.0 (15.4–59.2)

Sioma	4	0	0	0	0	0

**Table 2:** Prevalence of LPBE results, mixed infection, and PrioCHECK FMDV-NS test results in relation to age.

Age category	*n*	Prevalence (95% CI)—LPBE	Mixed infection	Prevalence (95% CI)—PrioCHECK FMDV-NS
Less than 1 year	4	0	0	0
1-2 years	37	100 (100-100)	64.9 (57.6–72.2)	100 (100-100)
3-4 years	49	93.9 (87.2–100)	87.7 (78.1–97.4)	85.7 (75.9–95.5)
5-6 years	9	100 (100-100)	88.9 (87.1–90.7)	100 (100-100)
*p* value		0.413	0.497	0.451

**Table 3:** Prevalence of LPBE results, mixed infection, and PrioCHECK FMDV-NS results in buffalo according to sex category.

Sex	*n*	Prevalence (95% CI)—LPBE			Mixed infection	Prevalence (95% CI)—PrioCHECK FMDV-NS
		SAT1	SAT2	SAT3		
Female	61	59 (46.7–71.0)	75 (64.1–85.9)	24.5 (13.7–35.2)	76.5 (64.5–88.5)	86.9 (78.4–95.4)
Male	38	63.2 (47.9–78.5)	71.1 (56.7–65.5)	28.9 (14.5–43.3)	62.4 (54.9–69.9)	81.6 (69.3–93.9)
*p* value		0.861	0.278	0.778	0.533	0.567

**Table 4:** LPBE test and PrioCHECK FMDV-NS test cross-tabulation.

	PrioCHECK FMDV-NS test		
	Negative	Positive	Total
LPBE test			
Negative	4	3	7
Positive	11	81	92
Total	15	84	99
